# Grammatical Gender in American Norwegian Heritage Language: Stability or Attrition?

**DOI:** 10.3389/fpsyg.2016.00344

**Published:** 2016-03-16

**Authors:** Terje Lohndal, Marit Westergaard

**Affiliations:** ^1^Department of Language and Literature, Norwegian University of Science and TechnologyTrondheim, Norway; ^2^Department of Language and Culture, University of Tromsø The Arctic University of NorwayTromsø, Norway

**Keywords:** acquisition, American Norwegian, declension class, gender, heritage language

## Abstract

This paper investigates possible attrition/change in the gender system of Norwegian heritage language spoken in America. Based on data from 50 speakers in the Corpus of American Norwegian Speech (CANS), we show that the three-gender system is to some extent retained, although considerable overgeneralization of the masculine (the most frequent gender) is attested. This affects both feminine and neuter gender forms, while declension class markers such as the definite suffix remain unaffected. We argue that the gender category is vulnerable due to the lack of transparency of gender assignment in Norwegian. Furthermore, unlike incomplete acquisition, which may result in a somewhat different or reduced gender system, attrition is more likely to lead to general erosion, eventually leading to complete loss of gender.

## Introduction

In his seminal study, Corbett (1991, p. 2) states that “[g]ender is the most puzzling of the grammatical categories.” It involves the interaction of several components: morphology, syntax, semantics, phonology, as well as knowledge about the real world. Languages also differ in terms of how many (if any) genders they have. This means that gender is a property of language which must be inferred from the input to which both child and adult learners of a language have to be finely attuned.

We follow Hockett (1958, p. 231) in defining gender as follows: “Genders are classes of nouns reflected in the behavior of associated words.” This means that gender is expressed as agreement between the noun and other elements in the noun phrase or in the clause and that affixes on the noun expressing e.g., case, number or definiteness are not exponents of gender (Corbett, 1991, p. 146). We refer to the marking on the noun itself as an expression of declension class (cf. Enger, 2004; Enger and Corbett, 2012; see also Kürschner and Nübling, 2011 for a general discussion of the difference between gender and declension class in the Germanic languages). This has an interesting consequence for the definite article in Norwegian, which is a suffix (more on this below). A distinction is also commonly made between gender assignment and gender agreement. Gender assignment is what is typically referred to as an inherent property of the noun, e.g., *bil*_(*M*)_ “car” and *hus*_(*N*)_ “house,” while gender agreement refers to agreement on other targets that is dependent on the gender of the noun, e.g., the indefinite articles and adjectives in *en*_.*M*_ *fin*_.*M*_ *bil*_(*M*)_ “a nice car” and *et*_.*N*_ *fint*_.*N*_ *hus*_(*N*)_ “a nice house”^1^. The literature also differentiates between lexical vs. referential gender (Dahl, 2000), or in the terminology of Corbett (1991), syntactic vs. semantic gender. The former refers to the inherent and invariable gender of a noun, e.g., *papa* “daddy” in Russian, which is always masculine, whereas the other refers to cases where gender depends on the referent, e.g., *vrac* “doctor,” which may take either feminine or masculine agreement.

In this article, we provide a case study of gender assignment in a population of heritage speakers of Norwegian who have lived their entire lives in America, often without ever visiting Norway. We follow Haugen (1953) in referring to this variety as American Norwegian, and here we study whether the use of gender differs in any way from the traditional use of gender in Norwegian dialects. We are also interested in the nature of possible discrepancies. This will provide important information on how gender systems may change over time, especially in contexts with reduced input and use, and we compare the situation in American Norwegian to heritage Russian spoken in the US. As Polinsky (2008, p. 40) emphasizes, “[s]ince very little is actually known about heritage language speakers, studying different aspects of language structure in this population is important.” The current paper contributes to this end in that it provides an additional investigation into the linguistic structure of heritage languages.

The structure of the paper is as follows. In the next section, we introduce gender and its manifestations within the Norwegian noun phrase. We then outline some relevant background from acquisition and heritage contexts, and the following section introduces our research questions, participants, and methodology. We then present our results, followed by a discussion and some concluding remarks.

## Gender and the norwegian noun phrase

Norwegian dialects traditionally distinguish between three genders: masculine, feminine and neuter. While many languages with gender have reliable morphophonological gender cues, e.g., Spanish or Italian (where a noun ending in –*o* marks masculine and –*a* marks feminine), gender assignment in Norwegian is non-transparent. That is, from just hearing a noun, e.g., *bil* “car,” *bok* “book,” or *hus* “house,” a learner cannot make out its gender. It is only when nouns appear with associated words that the gender can be identified, e.g., the indefinite article, as in *en.**n* *bil*_(*M*)_*, ei.f bok*_(*F*)_, and *et.n hus*_(*N*)_. Nevertheless, Trosterud (2001) proposes 43 different assignment rules and argues that they may account for 94% of all nouns in the language. These assignment rules include three general rules, nine morphological rules, three phonological rules, and 28 semantic rules. However, each rule has numerous exceptions, making it less clear if or how this rule-based account could actually predict gender in acquisition situations. Thus, we follow Rodina and Westergaard (2013, 2015a,b) in assuming that the acquisition of gender in Norwegian is opaque and must be learned noun by noun. This makes Norwegian gender a challenging property to acquire in a heritage language situation, where there is typically reduced input (see O'Grady et al., 2011).

Norwegian has two written standards, *Nynorsk* and *Bokmål*, the latter being by far the dominant one (see Venås, 1993 for more information about the Norwegian language situation). In *Bokmål*, all feminine nouns may take masculine agreement, which means that this written variety may use only two genders, common and neuter. The historical reason for this is that *Bokmål* is a development of the Danish written standard, and in Danish (as well as in Swedish and Dutch) the gender system has been reduced from one that distinguished three genders to one that generally only has two. The three-gender system has generally been retained in spoken Norwegian, in virtually all dialects (except Bergen and parts of Oslo). However, some recent studies indicate that a change from a three-gender system to a two-gender system is underway in the Tromsø dialect (Rodina and Westergaard, 2015a). More about this below.

Norwegian noun phrase syntax is relatively complex, and it has been extensively discussed in the literature; see Delsing (1993), Vangsnes (1999), and Julien (2005). Here we only discuss aspects of the noun phrase that are relevant for gender. Norwegian dialects also differ considerably with respect to the specific morphological marking on nouns. Table 1 provides an overview of the three-way gender system (based on the written *Bokmål* norm).

**Table 1:** **The traditional three-gender system of Norwegian**.

**Gender**	**Masculine**	**Feminine**	**Neuter**
Indefinite	**en** hest *a horse*	**ei** seng *a bed*	**et** hus *a house*
Definite	hest**en** *horse*.def**	seng**a** *bed*.def**	hus**et** *house*.def**
Double	**den** hest**en**	**den** seng**a**	**det** hus**et**
definite	*that horse*.def**	*that bed*.def**	*that house*.def**
Adjective	en **fin** hest	ei **fin** seng	et **fint** hus
	*a nice horse*	*a nice bed*	*a nice house*
Possessive	**min** hest/hest**en**	**mi** seng/seng**a mi**	**mitt** hus/hus**et mitt**
	**min**	*my bed/bed.def my*	*my*
	*my hourse/house.def*		*my horse/horse.def*
	*my*		*my*

Gender in Norwegian is mainly expressed inside the noun phrase (and on predicative adjectives, not discussed in this article). Thus, gender is marked on the indefinite article, e.g., *en* “a.m,” *ei* “a.f,” and *et* “a.n,” and on adjectives, where we find syncretism between M and F forms^2^.

As shown in Table 1, the definite article in Norwegian is a suffix, e.g., *hesten* “the horse,” *senga* “the bed,” *huset* “the house.” Some traditional grammars of Norwegian analyze the post-nominal definite suffix as an expression of gender (e.g., Faarlund et al., 1997), mainly because it is derived diachronically from postnominal demonstratives (separate words), which used to be marked for gender. Given our definition in the Introduction, however, these suffixes do not express gender, but should be considered to be declension class markers.

Since the definite suffix is sometimes considered to express gender, also in current work (e.g., Johannessen and Larsson, 2015), it is worth pausing to consider the evidence in favor of suffixes being declension class markers. This view is most prominently articulated by Lødrup (2011), based on a careful investigation of (a variety of) the Oslo dialect, where the feminine gender is argued to have been lost. The main piece of evidence is that despite the –*a* suffix (definite article) appearing on previously feminine nouns, all associated words are inflected as masculine in this dialect. Thus, the pattern is *en bok* “a.m book,” but *boka* “the book” (with the definite suffix for feminines). All adjectives and possessives are masculine, with the exception of certain instances of postnominal possessives. Together, these facts indicate that the gender of these nouns is M and that the suffix is indicating something that is not gender. Lødrup (2011), following Enger (2004), argues that the suffix expresses declension class, the inflection that is used for definite forms. As Alexiadou (2004, p. 25) points out, “[…] inflection class […] is never relevant for the purposes of agreement. It merely groups nouns into classes, which do not determine any further properties.” In essence, then, the distinction between gender markers and declension class markers is based on different properties: The latter is always a bound morpheme and appears on the noun itself, whereas the former do not appear on the noun. Following Corbett and Fedden (2015), it could be argued that in systems where gender markers and declension class markers align, we have a *canonical* gender system, whereas the Oslo dialect exhibits a non-canonical gender system, where the definiteness suffix does not encode gender.

Gender is also marked on possessives, which may be either pre- or post-nominal. Note that the noun is marked for definiteness when the possessor appears after the noun. In contrast, the definite suffix is impossible if the possessor is prenominal. According to Anderssen and Westergaard (2012), who have investigated both the NoTa corpus of adult speech (Oslo)^3^ as well as a corpus of child-directed speech recorded in Tromsø (Anderssen, 2006), the frequency of the postnominal possessor construction is much higher than the prenominal one (attested approximately 75%). The proportion of the postnominal possessor construction has been found to be even higher in American Norwegian heritage language, as the majority of the speakers investigated (*N* = 34) produce virtually only this word order (Westergaard and Anderssen, 2015). This is relevant for our investigation of gender, as it has been argued that the possessor is not an exponent of gender when it is placed postnominally (cf. Lødrup, 2011). This means that it could be treated like a declension class marker just like the definite suffix, and as just mentioned, the postnominal possessive also retains the feminine form much more than the prenominal one. We return to this in the Section Our study: Participants, Hypotheses and Methodology.

Finally, we should note that Norwegian exhibits a phenomenon called double definiteness, requiring that definiteness be marked twice in certain contexts, notably in demonstratives and in modified noun phrases. This means that definiteness is marked both on a pre-nominal determiner and on the suffix. While double definiteness adds complexity to the Norwegian noun phrase, it is also worth noting that in case of the prenominal determiner, there is again syncretism between M and F forms (cf. Table 1).

## Grammatical gender in acquisition and attrition

### The acquisition of gender

Grammatical gender is a complex linguistic phenomenon. A child or a second language learner acquiring a language with gender thus often has to internalize a range of different cues that contribute to determining the gender of a given noun. For the acquisition of grammatical gender in Norwegian, the lack of transparency of gender assignment has been shown to be a major challenge. While gender is typically acquired around the age of three in languages with a transparent gender system, such as Russian (e.g., Gvozdev, 1961) or many Romance languages (e.g., Eichler et al., 2012, on various bilingual Romance-German combinations), gender has been shown to be in place relatively late in Norwegian. Based on corpora of two monolingual and two bilingual (Norwegian-English) children (age approximately 2–3), Rodina and Westergaard (2013) found considerable overgeneralization of masculine forms (by far the most frequent forms in the input) to both feminine and neuter nouns (63 and 71% respectively). In a more recent experimental study of somewhat older children and adults, Rodina and Westergaard (2015a) find that neuter gender is not in place (at 90% accuracy; cf. Brown, 1973) until the age of approximately 7. It is also shown that the feminine is even more vulnerable among the older children. Rodina and Westergaard argue that this latter finding is due to an ongoing change in the dialect (Tromsø) from a three-gender system to a two-gender system, common and neuter. In both studies, they also show that, while proper gender forms such as the indefinite article are late acquired, the corresponding declension class markers (e.g., the definite suffix) are target-consistently in place from early on. In fact, the acquisition pattern for indefinite and definite forms are the mirror image of one another at an early stage, with non-target-consistent production around 90% for the former category and only about 10% for the latter. This means that young children typically produce the masculine form of the indefinite article with nouns of all three genders (e.g., *en.M hest*_(M)_ “a horse,” *en.*m *seng*_(*F*)_ “a bed,” *en.**m* *hus*_(*N*)_ “a house,” cf. Table 1), while the definite suffix is target-consistent (*hest****en*** “the horse,” *seng****a*** “the bed,” *hus****et*** “the house”). Results confirming this pattern are also attested in an experimental study of bilingual Norwegian-Russian children (Rodina and Westergaard, 2015b). These findings show that learners do not create an immediate link between the definite suffix and the agreement forms, indicating that the two belong to different systems and thus support the distinction between gender and declension class in Lødrup (2011).

### Gender in heritage language situations

Over the past 20 years, there has been an increasing focus on the language of heritage speakers. We adopt the following definition of a heritage language: “A language qualifies as a heritage language if it is a language spoken at home or otherwise readily available to young children, and crucially this language is not a dominant language of the larger (national) society (Rothman, 2009, p. 156; see also e.g., Rothman, 2007; Polinsky, 2008; Benmamoun et al., 2013). One characteristic of heritage grammars is that they may be different from that of speakers acquiring the same language as a majority language due to incomplete acquisition (e.g., Polinsky, 1997, 2006; Montrul, 2002, 2008; Sorace, 2004; Tsimpli et al., 2004) or attrition (e.g., Pascual y Cabo and Rothman, 2012; Putnam and Sánchez, 2013). That means that a heritage language grammar may represent a change compared to the grammar of the previous generation as well as the relevant non-heritage variety.

The baseline language for a heritage speaker is the language of exposure during childhood. This means that a heritage speaker of Russian in the US should not strictly speaking be compared to a speaker of Russian in Russia. This makes studying heritage languages quite challenging, given that it is often difficult to establish the relevant properties of the primary linguistic data that the learners have been exposed to. Due to this lack of data across generations, a comparison is often made between the heritage language grammar and the non-heritage variety—with the caveat that the latter does not necessarily represent the input to the generation of heritage speakers studied. This is what we have had to do in the current study. Heritage speakers also differ from non-heritage speakers of the same language with respect to the amount of variation attested in their production; while some speakers have a fairly stable grammar, others display a more variable grammar, not applying rules consistently (see Montrul, 2008 for discussion).

It is well known that for heritage speakers, the amount of input and use of the language during childhood varies (see Montrul et al., 2008, among many others). Given the complexity of gender, it is to be expected that heritage speakers face difficulties with this part of the grammar. This has been investigated for Russian heritage language in the US by Polinsky (2008). Like Norwegian, Russian has three genders: masculine, feminine, and neuter; see Corbett (1991, pp. 34–43) and Comrie et al. (1996, pp. 104–117) for further details and references. According to Corbett (1991, p. 78) the distribution of the three genders is M 46%, F 41%, and N 13%. Gender agreement is marked on adjectives, participles, demonstratives, possessive pronouns, past tense verbs and some numerals, and gender assignment is relatively transparent in that M nouns typically end in a consonant, F nouns in –*a*, and N nouns in –*o*. There are also some classes of nouns with non-transparent gender assignment.

Given somewhat reduced input, heritage speakers are typically exposed to fewer cues for gender assignment than children learning non-heritage Russian. Polinsky (1997, 2006) shows that less proficient American Russian speakers do not fully master the complex system of declension classes. In Polinsky (2008, p. 55), she demonstrates that two new gender systems have developed among the heritage speakers, both somewhat different from that of the non-heritage variety: (1) a three-gender system used by the more proficient speakers, differing from the non-heritage variety in that opaque N nouns ending in an unstressed –*o* are produced with F gender (i.e., they are pronounced with a schwa and therefore confused with the feminine ending –*a*), and (2) a two-gender system produced by the less proficient speakers where all N nouns have migrated to F. It is speculated that the latter speakers do not master the complex system of declensional case endings, and in the absence of this knowledge, they are relying on a purely phonological cue, i.e., whether the noun in its base form (Nominative singular) ends in a consonant or a vowel. The two systems are described in (1)–(2).

1. More proficient speakers: Three-gender system
  - nouns ending in a consonant are M
  - nouns ending in a stressed –*o* are N
  - all other nouns are F (i.e., including nouns ending in an unstressed –*o*, which are N in non-heritage Russian)
2. Less proficient speakers: Two-gender system
  - nouns ending in a consonant are M
  - nouns ending in a vowel are F

In a recent study of Norwegian-Russian bilingual children growing up in Norway (age 4–8), Rodina and Westergaard (2015b) find an even more reduced gender system in some of the children. The amount of input is argued to be crucial: While children with two Russian-speaking parents are virtually identical to monolingual children growing up in Russia, the bilinguals with the least amount of input (only one Russian-speaking parent who does not use Russian consistently with the children) have considerable problems with gender, not just the opaque nouns, but also the transparent ones. In fact, some of these children produce almost exclusively masculine forms, overgeneralizing them to feminine nouns 77% and to neuters as much as 94%, which means that they do not seem to have any gender distinctions at all. Since these children are only up to 8 years of age, follow-up studies are necessary in order to find out whether they will eventually converge on the target, or whether they are developing a Russian heritage variety without gender.

### Gender and diachronic change

It is well known that M and F genders have collapsed into common gender (C) in many Germanic languages and dialects. This change has taken place e.g., in Dutch, Danish, and the Bergen dialect of Norwegian (Jahr, 1998; Nesse, 2002; Trudgill, 2013). Furthermore, Conzett et al. (2011) have attested a similar change in certain dialects in North Norway (Kåfjord and Nordreisa). This region has had extensive language contact with Saami and Kven/Finnish, languages which do not have grammatical gender. This language contact is argued to have caused a reduction of the gender system of the Norwegian spoken in this area from three to two (C and N). At the same time the declension system is intact. This means that while the feminine indefinite article *ei* “a.f” is virtually non-existent in the data, the corresponding definite suffix still has the –*a* ending typical of F nouns. This is illustrated in (3).

(3) a. en bok      - boka
      a.c book.c        - book.f.def

This pattern is identical to what Lødrup (2011) found for Oslo speech (cf. the Section Gender and the Norwegian Noun Phrase above). The cause of the change in Oslo is generally argued to be sociolinguistic: The *Bokmål* written standard allows the use of only two genders, and a spoken version of this variety enjoys a high social prestige in certain speaker groups. Thus, the three-gender system of the traditional dialects has gradually become associated with something rural and old-fashioned. The pattern attested means that a reduced gender system has developed in both areas (common and neuter), but at the same time a more complex declension system, in that the new common gender has two declension classes in the definite form, i.e., *en bil–bil****en*** “a car–the car” and *en bok–bok****a*** “a book–the book.”

Even more recent research is providing us with data on a real-time case of language change. Based on an experimental study, Rodina and Westergaard (2015a) demonstrate that F gender is rapidly disappearing from the speech of children and young adults in Tromsø: The F indefinite article is replaced by M, yielding common gender, but as in Oslo and Kåfjord/Nordreisa, the definite suffix is still preserved in its F form. Note that this pattern is also identical to what has been attested in early Norwegian child language (cf. the Section The Acquisition of Gender). While Rodina and Westergaard (2015a) also assume that the cause of this change is sociolinguistic, they argue that the *nature* of the change is due to acquisition: While the N forms are saliently different from the other two genders, there is considerable syncretism between M and F (e.g., adjectives and prenominal determiners), making it more difficult to distinguish the two in the acquisition process (cf. Table 1). Furthermore, while the real gender forms are very late acquired (around age 5–7), the declensional suffixes are target-consistently in place very early (around age 2), cf. Anderssen (2006) and Rodina and Westergaard (2013). Thus, the late acquired forms are the ones that are vulnerable to change.

The three studies briefly presented here demonstrate that F gender is disappearing or already lost from several Norwegian dialects. We would thus expect that F gender should be vulnerable in an acquisition context where there is somewhat reduced input, e.g., in a heritage language situation. In the following sections, we present our study of gender in American Norwegian.

## Our study: participants, hypotheses, and methodology

### Norwegian heritage language in america

According to Johannessen and Salmons (2012, p. 10), Norwegian immigration started in 1825, when the first Norwegians arrived in New York. By 1930, as many as 810,000 people had arrived in the US and an additional 40,000 in Canada. In the US, they settled mostly in the Midwest, predominantly in the Dakotas, Illinois, Iowa, Minnesota, and Wisconsin. The Norwegians built churches and schools and also had their own newspapers, *Decorah-Posten* and *Nordisk Tidende*. According to Johannessen and Salmons (2012, p. 6) 55,465 people reported Norwegian as their home language in the 2000 US Census. However, most of the current heritage speakers are above 70 years of age. American Norwegian as a heritage language can thus be said to be in its final stages (cf. Johannessen and Salmons, 2012).

American Norwegian was first documented and studied by Haugen (1953), based on fieldwork in the late 1930s and 1940s and subsequently, this heritage language was studied by Hjelde (1992, 1996). More recently, extensive fieldwork has been conducted in connection with the NorAmDiaSyn project, and data have been collected from a number of 2nd to 4th generation immigrants who learned Norwegian as their L1 from parents and grandparents. According to Haugen (1953, p. 340), the first immigrants were from the west coast of Norway, but around 1850, large numbers came from rural Eastern parts of Norway (Johannessen and Salmons, 2015, p. 10). It is mainly these Eastern varieties that are spoken today: Johannessen and Salmons (2015) remark that in 2010 it was difficult to find speakers of western dialects. For most of the immigrants, there was little or no support for Norwegian language in the community. Consequently, these speakers have generally been bilingual since the age of 5–6, and they have been dominant in English since this time. The background information offered about the corpus participants is relatively sparse: Year of birth, language of schooling and confirmation, literacy in Norwegian, number of visits to Norway as well as other contact with the country. In addition, we know which generation immigrant they report to be, and for some of them, the year their family arrived in the US. There is no information about the amount of use of Norwegian in adulthood. The language of schooling is English for all of them (except two informants for which this information is missing), and the large majority (43/50) had their confirmation in English. Contact with Norway varies between “some” and “often,” and many have never visited the country. Typically, these heritage speakers have never had any instruction in Norwegian, and most of them have no literacy skills in the language.

The majority of the participants are between 70 and 100 years old today, and as they have not passed on the language to the next generation, they do not have many people to communicate with in Norwegian. Thus, most of these heritage speakers hardly ever use Norwegian any more, and at the time of the CANS recordings, many of the participants had not uttered a word of Norwegian for years, one participant for as long as 50 years. The initial impression of their Norwegian proficiency is that it is quite rusty, but once these speakers warm up, many properties of the language turn out to be intact (Johannessen and Laake, 2015). Given the language profile of these learners (monolingual Norwegian speakers until school age, predominantly English dominant in adult life, and hardly using Norwegian at all in old age) it is possible that any discrepancies between their language and the non-heritage variety should be due to attrition rather than incomplete acquisition.

So far, data from 50 informants have been transcribed and now make up the Corpus of American Norwegian Speech (CANS) (Johannessen, 2015). This corpus consists of speech data collected through interviews (by an investigator from Norway) and conversations among pairs of heritage speakers. Each recording lasts approximately a half hour to an hour, meaning that there is relatively sparse data per informant.

### Hypotheses and predictions

Based on the properties of the gender system of Norwegian and previous research on gender in acquisition and change, we formulate the following hypotheses and predictions for American Norwegian:

(4) Hypotheses
  - Gender is vulnerable in American Norwegian
  - Gender forms and declensional suffixes behave differently
  - F is more vulnerable than N due to syncretism with M
(5) Predictions
  - Speakers will overgeneralize M gender forms
  - Declensional suffixes will be retained
  - F will be affected first; i.e., (some speakers of) American Norwegian will have a two-gender system (common and neuter)

We expect gender to be vulnerable in a situation with reduced input such as Norwegian heritage language, especially given the non-transparency of the gender system and the relatively late acquisition attested by Rodina and Westergaard (2015a). We also expect to see a difference between forms that express gender proper (i.e., agreement) and the declensional endings, which has been attested in previous research on both acquisition and change (e.g., Lødrup, 2011; Rodina and Westergaard, 2013). Finally, as in Russian heritage language and in many Germanic varieties, we may also see reductions in the gender system, either from a three- to a two-gender system (common and neuter) or to a system where gender breaks down completely.

### Methodology

We have used CANS to probe the usage of gender in American Norwegian. We have generally excluded English loan words appearing with gender marking (see Flom, 1926; Hjelde, 1996; Nygård and Åfarli, 2013; Alexiadou et al., 2015 on this issue)^4^. Our main focus here is on gender assignment, and we have therefore also disregarded agreement between different gender forms within the nominal phrase. We have searched CANS for the following forms:

(6) a. the indefinite article followed by a noun (occasionally with an intervening adjective)
     b. possessives
     c. definite forms

We have also compared the data from the CANS corpus to a sample of the Nordic Dialect Corpus (Johannessen et al., 2009). This allows us to compare the gender system of American Norwegian to that of contemporary Norwegian. We would like to emphasize that we obviously do not assume that the heritage speakers recorded in the CANS corpus were exposed to a variety of Norwegian that is identical to the non-heritage variety spoken today. But we are interested in investigating possible changes in the heritage variety, possibly across several generations, and these are the data we have available to make the comparison. We have used the part of the Nordic Dialect Corpus which covers the dialects spoken in the Eastern part of Norway (excluding the capital, Oslo), the area from which most of the ancestors of the heritage speakers originate. The Nordic Dialect Corpus consists of structured conversations between speakers of the same dialect and as such, the two corpora are comparable with respect to the recording situations. In the Nordic Dialect Corpus, speakers are classified as either “old” (over 50) or “young” (under 30), where most of the informants in the two groups are in their 60s and 20s respectively. The corpus was recorded between 2008 and 2011.

Both corpora have been transcribed into a dialect version and a standardized *Bokmål* transcription. The corpora are tagged, and the transcriptions are directly linked to the recordings. In CANS, we found that in several cases, the *Bokmål* transcription had standardized the gender according to the *Bokmål* official dictionary, even when the informants actually used a different gender. Thus, we have had to check the recordings carefully in order to be sure that we had reliable transcriptions. We generally did not find errors in the dialect version (corresponding to the pronunciation), which made us trust that this transcription is sufficiently correct for our present purposes. Furthermore, there are some instances where the F indefinite article has been transcribed simply as /e/. We have listened to all of these and in all cases the informants seem to be saying the feminine form /ei/. They have therefore been counted as occurrences of the F indefinite article.

Compound nouns (e.g., *skolehus* “school house”) have been counted separately. In Norwegian, the right-hand part of the compound is always the head noun and thus determines the gender. For several of the compound words in the corpus, the right-hand noun also occurs independently (e.g., *hus* “house”). Instances where the noun was not uttered completely were disregarded. In cases where speakers correct themselves as in (7a), we only counted the latter form. Examples have also been counted if they occur in what would be considered an ungrammatical or unidiomatic structure in Norwegian, e.g., (7b), which is presumably a direct translation of an English expression.

(7) a. *ei # en familie*             (flom_MN_02gm)
     a.f # a.m family.m
     b. *vi hadde en god tid*      (portland_ND_01gm)
          we had a.m good time.f
          Target form (intended meaning): *vi hadde det morsomt*
            [lit.: we had it fun]

With these methodological considerations in mind, let us move on to the results of our study.

## Results

### Gender marking on the indefinite article—overall results

Our search in CANS first of all revealed that all three gender forms are attested in the data. Examples illustrating the use of the three indefinite articles *en, ei*, and *et* (M, F, and N) are provided in (8)–(10). In these examples, the gender marking is entirely in line with what we would expect in present-day non-heritage Norwegian. It is also worth noticing that although there is some language mixing between English and Norwegian here, the sentences are predominantly Norwegian in structure and lexicon.

(8) *vi kjøpte **en butikk***           (blair_WI_04gk)
       we bought a.m store(m)
       “we bought a store”
(9) *og **ei uke**      sia så h- visita vi parken      i   Blair*
       and a.f week(f) ago so visited   we park.def in Blair
       *her*                                                        (blair_WI_01gm)
       here
       “a week ago we visited the park in Blair here”
(10) *we got har bare **et   tre***        (coon_valley_WI_04gm)
       we got have only a.n tree(n)
       “we only got one tree”

In a study of the *Nynorsk* dictionary (Hovdenak et al., 1998), which is the written norm that is closest to the contemporary dialects, Trosterud (2001) has found that out of the 31,500 nouns listed there, 52% are M, 32% are F, and 16% are N. These numbers are somewhat different from the distribution in the spoken language. Rodina and Westergaard (2015a) have investigated proportions of the indefinite article in a corpus of child and child-directed speech recorded in the mid-90s (Anderssen, 2006) and found that M forms are even more frequent in the input than in the dictionary, 62.6%, while the F and N forms are more or less equally represented, 18.9 and 18.5% respectively (*N* = 2980). We have investigated the occurrences of the three indefinite articles in the Nordic Dialect Corpus, and we find that the distribution in the data of the “old” speakers is virtually identical to Rodina and Westergaard's (2015a) findings, see Table 2. In the data of the “young” speakers, on the other hand, the F indefinite article is only attested 5.4%, while the proportion of M forms has increased to 74.9%. We believe that it is likely that these numbers reflect an ongoing change involving the loss of F forms also in these dialects, just like in Oslo and Tromsø (cf. the Section Gender and Diachronic Change). A careful study of the Nordic Dialect Corpus in order to confirm (or disconfirm) this hypothesis has to be left for future research.

**Table 2:** **Token distributions of the three indefinite articles *en* (M), *ei* (F) and *et* (N), in CANS and in Eastern Norwegian dialects (Nordic Dialect Corpus)**.

**Gender**	**CANS (*N* = 50)**	**NorDiaCorp (old, *N* = 127)**	**NorDiaCorp (young, *N* = 66)**
M	76.3% (753)	64.8% (1833)	74.9% (909)
F	16.9% (165)	18.2% (514)	5.4% (66)
N	6.9% (67)	17.0% (481)	19.7% (239)

In Table 2, we have also provided the relevant counts from the CANS corpus. Overall, the figures for the heritage speakers indicate that gender is relatively stable in American Norwegian, as they are quite similar to the older speakers in the Nordic Dialect Corpus, except for a lack of neuter forms. However, a closer look reveals that the heritage speakers are overgeneralizing the M gender forms quite substantially to both F and N nouns. We now turn to a discussion of these discrepancies between the CANS corpus and forms found in present-day spoken Norwegian.

### Overgeneralization—indefinite articles

Although all gender forms are represented in the corpus, and gender thus appears to be relatively stable, there are several cases of what we will refer to as non-target-consistent forms, i.e., forms that are different from what would be expected in non-heritage Norwegian. When determining the gender of nouns in non-heritage Norwegian, we have used the *Nynorsk* Dictionary with some adjustments for differences between the dictionary and the gender typically found in Eastern Norwegian dialects^5^. In this section, we consider nouns with the indefinite article, either by itself or together with an adjective. We first consider all noun occurrences (tokens) and then the number of different nouns (types) appearing in the corpus.

In the corpus, we find 236 occurrences that are F nouns. As many as 39.0% (92/236) of these appear with M gender; see (11)–(13).

(11) *og    om    in  in **en uke**      da    så # kom han til byen igjen*
          and about in in a.m week.f then so came  he    to city   again
                                                               (rushford_MN_01gm)
(12) *og # tre brødre og     r- en s- # **en søster***
          and three brothers and a.m a.m sister.f
                                               (blair_WI_04gk)
(13) *ja # em # har du # har du **en ku** enda?*
         yes em   have you have you a cow still?
                                     (coon_valley_WI_01gk)

We should note that there is considerable variation between M and F forms used with some F nouns in the corpus. For example, *datter* “daughter” occurs both with F and M indefinite articles. Speakers appear to be consistent and typically do not alternate. However, given the sparse data in CANS, we very often find that a speaker only produces one or two instances of the same noun. For this reason, we cannot address the question of speaker consistency.

Turning to the neuter, we find 164 nouns which are N according to the *Nynorsk* dictionary and our Eastern Norwegian adjustments. Of these, as many as 48.8% (80/164) appear with the M indefinite article. Examples are provided in (14)–(16).

(14) *# bestemor # var født # hun var på **en fjell ##***
          grandma     was born she    was onm mountain.n
                                                         (chicago_IL_01gk)
(15) *fire   år #   og   en  **en  år**       at to the university*
          four years and a.m a.m year.f at to the university
                                               (wanamingo_MN_04gk)
(16) *fikk jeg **en pass***           (coon_valley_WI_02gm)
          got I a.m passport.n

There are also occasional N nouns appearing with F gender forms, 10.4% (17/164); see the examples in (17)–(19). Considering the current trend in Norway with F gender in the process of disappearing, it is rather surprising that there is overuse of feminine forms.

(17) *det var ei menneske*           (westby_WI_05gm)
            it  was a.f human.being.n
  (18) *han var her det var ei bryllup*
          he was here it was a.f wedding.n
                                (harmony_MN_02gk)
(19) *jeg tror ikke jeg sa ei eneste norsk        ord*
          I think not I said a.f single Norwegian word.n
                                                  (harmony_MN_04gm)

Finally, we found four examples of non-target-consistent gender on M nouns, in all cases produced with the F indefinite article. This amounts to only 0.7% (4/576).

We now take a closer look at the number of actual nouns involved (types). Due to the very low number of non-target-consistent M nouns, we only consider F and N. The list in (20) provides all F nouns that occur with the target-consistent indefinite article (altogether 51 nouns), where the ones in bold are sometimes produced with M (10 nouns). In (21) we find 21 F nouns that *always* appear with M gender in the corpus. In total, there are 72 different F nouns, of which 31 are either always or sometimes produced with M gender forms. This means that overgeneralization of types is 43.1% (31/72), which is similar to the frequency of noun tokens reported above, 39.0%.

(20) **F** = **F:** *stund* “time,” *søster* “sister” *kanne* “mug,” *trå* “yearning,” ***side*** “side,” ***kjerring*** “hag,” ***seng*** “bed,” ***uke*** “week,” *jente* “girl,” *lefse* “lefse,” ***kiste*** “coffin,” *mølle* “mill,” ø*ks* “ax,” ***tid*** “time,” *mjølking* “milking,” ***ku*** “cow,” *kvige* “heifer,” *grøft* “trench,” *brødpanne* “bread pan,” ***bok*** “book,” *trinse* “caster,” *mil* “mile,” *høstnatt* “fall night,” *datter* “daughter,” *dame* “lady,” *bjelle* “bell,” *tobakksseng* “tobacco bed,” ei *[female name removed]* “a female name,” *bestemor* “grandmother,” *hytte* “hut,” *frilledatter* “daughter of a mistress,” *gryte* “pot,” *aure* “trout,” *liste* “list,” *skrøne* “tall tale,” *rumpe* “butt,” *stikke* “peg,” *pakke* “package,” *pike* “girl,” *mor* “mother,” *trønderskrøne* “tall tale from Trøndelag,” *dør* “door,” *plattform* “platform,” *himmelseng* “four-poster bed,” ***kirke*** “church,” *tante* “aunt,” *hand* “hand,” *matte* “mat,” *lue* “cap,” *bøtte* “bucket,” ***datter*** “daughter” (41+10 = 51)
(21) **F-**>**M:** *blanding* “mixture,” *mil* “mile,” *flaske* “bottle,” *tale* “speech,” *stund* “while,” *gruppe* “group,” *ordbok* “dictionary,” *hast* “haste,” *rotte* “rat,” *vogn* “wagon,” *avis* “newspaper,” *pipe* “pipe,” *elv* “river,” *stripe* “stripe,” *kagge* “keg,” *purke* “sow,” *slekt* “family,” ø*y* “island,” *dialekt* “dialect,” *klasse* “class,” *lærerinne* “female teacher” (21)

Considering N nouns, (22) lists all the ones that occur with the target-consistent indefinite article (altogether 23 nouns). Nouns in bold also appear with M indefinite article (11 nouns), while nouns which are underlined also appear with F (8 nouns). In (23) we find N nouns which only appear with F indefinite article and in (24) N nouns that consistently appear with M indefinite article.

(22) **N** = **N: *hotell*** “hotel,” ***par*** “pair/couple,” **å*r*** “year,” *fat* “plate,” ***brev*** “letter,” *lass* “load,” *hus* “house,” *lag* “layer,” *hull* “hole,” ***skolehus*** “school,” ***bilde*** “picture,” *sted* “place,” ***fjell*** “mountain,” *blad* “magazine,” ***ord*** “word,” *rom* “room,” *leven* “noise,” ***stykke*** “piece,” *slag* “blow,” ***navn*** “name,” *minutt* “minute,” ***liv*** “life,” *problem* “problem” (12+11 = 23)
(23) **N-**>**F:** *menneske* “human being,” *hjem* “home,” *bryllup* “wedding,” ***barnebarn*** “grandchild,” *papir* “paper” (5)
(24) **N-**>**M:** *barnetog* “children's parade,” *farmeår* “farm year,” *program* “program,” *pass* “passport,” *tømmerhus* “log cabin,” *tog* “train,” *arbeid* “work,” *patent* “patent,” *dusin* “dozen,” *bord* “table,” *band* “band,” *lys* “light,” *oppstuss* “fuss,” *eiketre* “oak,” *utvandrermuseum* “emigration museum,” *kort* “card,” *mål* “measure,” *måltid* “meal,” *kupp* “bargain,” *selvfirma* “independent company,” *orkester* “orchestra” (21)

The total number of different N nouns is 49. As many as 34 of them (always or sometimes) appear with an M indefinite article (69.4%), while 13 (always or sometimes) appear with F gender (26.5%). This means that N nouns are quite unstable in the production of these heritage speakers.

Table 3 summarizes our findings, considering both the total number of noun occurrences (tokens) in the data as well as the number of different nouns (types).

**Table 3:** **Summary of noun tokens and noun types appearing with a non-target-consistent indefinite article**.

**Direction**	**Total number of examples (tokens)**	**Number of different nouns (types)**
F → M	39.0% (92/236)	43.1% (31/72)
N → M	48.8% (80/164)	69.4% (34/49)
N → F	10.4% (17/164)	26.5% (13/49)

### Gender vs. inflection class

As we have seen, many of the F and N nouns in the corpus (always or sometimes) occur with an M indefinite article (31/72 and 34/49 respectively), shown in (25) and (27). However, when we consider the definite suffixes on these same nouns, they are usually the feminine –*a* and neuter –*et* forms, not the masculine –*en*. This is shown in (26) and (28), where the numbers in parentheses indicate occurrences. In fact, for the neuter nouns, the masculine declensional suffix is unattested (cf. Johannessen and Larsson, 2015).

(25) ***en*** *datter* “a daughter,” ***en*** *tid* “a time,” ***en*** *kirke* “a church,”                    ***en*** *uke* “a week”
(26) *datter****a*** (24) - *datter****en*** (0), *tid****a*** (206) - *tid****en*** (13), *kirk****a*** (80)-                    *kirk****en*** (3), *uk****a*** (14) - *uk****en*** (0)
(27) *en år* ‘a year’, *en tog* “a train,” *en hus* “a house,” *en lys* “a light”
(28) å*r****et*** (31) – å*r****en*** (0), *tog****et*** (9) – *tog****en*** (0), *hus****et*** (60) – *hus****en***                    (0), *lys****et*** (3) – *lys****en*** (0)

This mirrors findings from other studies, showing that when the feminine gender is lost, the definite suffix is retained (e.g., Lødrup, 2011; Rodina and Westergaard, 2015a). This demonstrates that the affixal definite article clearly behaves differently from the free gender morphemes that agree with the noun, e.g., the indefinite article, not only in contexts of acquisition and change, as attested in previous research, but also in heritage language.

Related to this is the result of our search for possessives in the corpus. Recall from the Section Gender and the Norwegian Noun Phrase that possessives in Norwegian may appear both in prenominal and postnominal position, and that Westergaard and Anderssen (2015) reported that in Norwegian heritage language, the postnominal construction is the preferred one. First of all, our findings show that the possessives used in the corpus are mainly high frequency kinship terms (more than 90%) of the type illustrated in (29)–(30); thus, they may be rote-learned or memorized and not necessarily be the result of a productive system. We also find that numbers are very low for all possessives except the first person singular, and this is therefore the only result that is reported here (Table 4).

(29) a. *mor mi* (44)
            mother my
            “my mother”
b. *søstera mi*    (10)
       sister.def my
       “my sister”
c. *bestemora         mi* (4)
       grandmother.def my
       “my grandmother”
(30) a. *far          min* (102)
       father.def my
       “my father”
b. *bror          min* (36)
       brother.def my
       “my brother”
c. *mannen      min* (35)
       husband.def my
       “my husband”

**Table 4:** **Distribution of gender marking for first person possessives in CANS (*N* = 50)**.

**Gender form**	**Prenominal**	**Postnominal**	**Total**
M (*min* “my”)	87/96 (90.6%)	251/414 (60.6%)	338/510 (66.3%)
F (*mi* “my”)	0 (–)	126/414 (30.4%)	126/510 (24.7%)
N (*mitt* “my”)	9/96 (9.4%)	37/414 (8.9%)	46/510 (9.0%)

Compared to the results in Table 2, where the proportion of F indefinite articles was only 16.9%, it is a bit surprising that the proportion of F forms is as high as 24.7%. However, as we mentioned above, the postnominal possessor has been argued to be a declension class marker and not an exponent of gender (Lødrup, 2011). In this table, we also see that the prenominal possessives behave differently from the postnominal ones, in that the feminine form is attested relatively frequently as a declension class marker (30.4%), and not at all in the gender form (in prenominal position). This difference becomes even clearer when we consider whether the gender forms have been used target-consistently: In Table 5, the feminine forms are always produced with M gender in prenominal position (the gender form) but they are generally retained when occurring postnominally, where we only find occasional non-target forms (both M and N). The fact that the F form is retained postnominally fits well with Lødrup's (2011) analysis that postnominal possessors behave like declension markers on a par with the affixal F definite endings. Turning to N nouns, we see that they also tend to migrate to M, somewhat more in prenominal than postnominal position (30.8 vs. 19.2%). In comparison, the masculine is virtually always produced with target-consistent gender agreement.

**Table 5:** **Distribution of genders for first person possessives in CANS (*N* = 50), target and non-target-consistent forms**.

**Gender**	**Prenominal**		**Postnominal**	
	**Target**	**Non-target**	**Target**	**Non-target**
M	40/40	0	226/228	2 (to N, 0.9%)
F	0	43 (to M, 100%)	126/137	7 (to M, 5.1%)+4 (to N, 2.9%)
N	9/13	4 (to M, 30.8%)	21/26	5 (to M, 19.2%)
TOTAL	49	47	373	18

### Individual results

The individual production results of each of the 50 participants in the corpus are provided in the Appendix, for the indefinite article only, as this is the most frequent form produced. As expected, there is a very limited amount of data per informant, so that it is impossible to provide complete profiles of the gender system of each of them. Nevertheless, the participants have been divided into four groups. In Group 1, there are four participants for which no conclusions can be drawn, as the production is too limited (one participant produces no indefinite forms at all and three participants only produce masculine forms—for masculine nouns). In Group 2, we find five participants who may possibly have an intact three-gender system, as they make no mistakes. However, each of them produces so few examples (11, 13, 9, 6, 6 respectively), and it is therefore possible that this is simply the result of sheer luck in the recording situation. Furthermore, only two of these five produce nouns in all three genders, while the remaining three only produce masculine and feminine nouns, not a single neuter. At the other end of the scale, there are nine informants who may not have gender at all (Group 3). These speakers produce masculine forms only, either for nouns belonging to two of the genders (four participants) or all three (five participants). The final group (Group 4) thus contains the majority of informants (32), who produce a mixture of forms. For these, target-consistency varies considerably, from participants making only one mistake (e.g., decorah_IA_01gm), who are thus similar to Group 2, to those who produce only one form that is not masculine (e.g., portland_ND_02gk) and are thus similar to Group 3. There is also variation with respect to which gender is more vulnerable, as some seem to have more problems with feminine nouns (e.g., webster_SD_02gm) and others with the neuter (e.g., coon_valley_WI_06gm), while others again have problems with both (e.g., stillwater_MN_01gm). Eight informants produce no feminine forms, which at first sight could indicate that they have a two-gender system consisting of common and neuter. However, two of them do not produce any feminine nouns at all, and all of them also make a considerable number of mistakes with the neuter. Thus, not a single informant displays a clear two-gender system where the neuter is intact and the feminine has merged with the masculine into common gender.

## Discussion

We now return to our hypotheses and predictions, repeated in (31)–(32) for expository convenience.

(31) Hypotheses
  - Gender is vulnerable in American Norwegian
  - Gender forms and declensional suffixes behave differently
  - F is more vulnerable than N due to syncretism with M
(32) Predictions
  - Speakers will overgeneralize M gender forms
  - Declensional suffixes will be retained
  - F will be affected first; i.e., (some speakers of) American Norwegian will have a two-gender system (common and neuter)

In the results section Gender Marking on the Indefinite Article–Overall Results, we saw that all the three genders are represented in the corpus, and the total numbers give the impression of a fairly stable system. However, when we considered the data in more detail (Section Overgeneralization—Indefinite Articles), we saw that there is considerable overgeneralization of M forms of the indefinite article to both F and N nouns (cf. Table 3). The substantial overgeneralization of M to F is unsurprising, given the findings from previous studies. However, in the present study there is clearly more overgeneralization affecting neuter than feminine nouns, both when we consider the overall number of occurrences (tokens, 48.8 vs. 39.0%) and the number of different nouns affected (types, 69.4 vs. 43.1%), cf. Table 3. In the prenominal possessives, we find that the feminines are produced with masculine forms 100% and the neuters approximately 31%. Based on these results, we conclude that gender is in fact vulnerable in American Norwegian, and thus that our Hypothesis A has been confirmed. Likewise, we can confirm Prediction A: Although there are a number of cases where neuter nouns migrate to the feminine (10.4% of the total number of neuters (tokens) and 26.5% of the number of different nouns (types), cf. Table 3), it is clear that the general pattern found for non-target-consistent forms is overgeneralization of the masculine.

Turning to Hypothesis and Prediction B, we saw in the Section Gender vs. Inflection Class that the definiteness suffix behaves very differently from the indefinite article. While feminine and neuter indefinite articles are frequently produced with masculine forms, the definite suffix is always target-consistent in the neuter and mostly also in the feminine. This means that our findings confirm previous research both from acquisition and change (cf. Sections The Acquisition of Gender and Gender and Diachronic Change), where the same distinction has been attested. As mentioned above, we consider the indefinite article to be an exponent of gender, whereas the affix is analyzed as a declension marker. The different behavior of these two elements also in this population of heritage speakers clearly shows that gender forms are much more prone to change than declension markers. The different behavior of the prenominal and postnominal possessives (at least for feminine nouns) also indicates that there is a distinction between the two that may be related to gender (cf. Lødrup, 2011).

It should be noted here that our claim that gender is vulnerable in Norwegian heritage language runs counter to the conclusion reached by Johannessen and Larsson (2015). Based on an investigation of a selection of the 50 speakers in CANS, they argue that grammatical gender is *not* affected by attrition. The main reason for the two different conclusions is that, unlike us, Johannessen and Larsson (2015) do consider the definite suffix as a gender marker. And since the form of the suffix is generally retained, they consider this evidence that gender is intact. Furthermore, they find that complex noun phrases (determiner-adjective-noun) are much more prone to errors than simple ones (adjective-noun), with 18% (20/113) vs. 2% (1/58) target-deviant agreement. They argue that this shows that gender is unaffected by attrition, since it is target-consistent in simple noun phrases, and they account for the target-deviance in the complex ones as a result of processing difficulties. In our view, another explanation is also possible: Given that the number of noun types in the corpus is quite low and mainly consists of high-frequency nouns, we could argue that the simple noun phrases are more likely to be rote-learned and memorized as chunks than the more complex ones, which require a productive system of gender agreement. Since this is in the process of breaking down, the complex noun phrases display more errors.

We then turn to our final hypothesis and prediction (C) and the issue whether F gender is more vulnerable than N and whether we see changes or reductions in the gender system. As discussed above, this has been attested in Russian heritage language; both a reduction from a three- to a two-gender system (Polinsky, 2008) and possibly a breakdown of gender altogether (Rodina and Westergaard, 2015b). We also know that a reduction in the gender system has happened in many Germanic varieties and is currently taking place in certain Norwegian dialects (cf. the Section Gender and Diachronic Change), that is, a reduction from a three-gender system to a system with just two genders, common and neuter. As noted above, disappearance of also the neuter gender is not an unlikely scenario, given the non-transparency of the system and the late acquisition of this property of the Norwegian language. The gender system may be further weakened by the considerable lack of input and use in this heritage language situation. However, as shown in the previous section, we do *not* find any evidence of a two-gender system in the production of any these 50 speakers. Instead we see a general erosion across the whole gender system, with both feminine and neuter nouns migrating to the most frequent gender form, the masculine. In fact, the majority of the speakers (*N* = 32) behave in this way (Group 4). The end result of this will presumably be a complete breakdown of gender altogether; i.e., a system without gender distinctions. It is possible that this is already attested in the production of the nine speakers in Group 3, who produce only masculine forms.

We would like to speculate about the reasons for this development; i.e., (1) why is grammatical gender vulnerable in heritage language, (2) why are declension class suffixes stable, and (3) why do we not see evidence of a two-gender system the way we predicted? Our findings partly correspond to what has been found in acquisition and change, i.e., proper gender forms such as the indefinite article are late acquired and prone to change, while the declensional suffixes are early acquired and remarkably stable. But we do not find a two-gender system (common and neuter), which is attested in some children and which is also the result of changes that have taken place in certain varieties of Norwegian.

An obvious answer to the first question corresponds to the general account for the late acquisition of gender in Norwegian, viz. the non-transparency of gender assignment. A system where gender has to be learned noun by noun is crucially dependent on a considerable amount of input. Unfortunately, we do not know much about the input to these speakers in childhood, but it is not inconceivable that it was somewhat limited. Given that gender has been found not to be fully in place until around age 6–7 (Rodina and Westergaard, 2015a), which is the time when these speakers experienced a language shift, it is possible that this property is the result of incomplete acquisition (e.g., Montrul, 2008). However, given the general profile of these heritage speakers mentioned above (monolingual Norwegian speakers until school age, English dominant in their adult lives, and hardly using Norwegian at all in old age), it is more likely that whatever discrepancies we find between their language and the non-heritage variety is due to attrition. This is further supported by the fact that there is considerable variation among these speakers. If this is the case, then we may speculate on a possible difference between incomplete acquisition and attrition with respect to gender: While the former process typically results in a systematic reduction in the gender system (e.g., from three to two genders), the latter affects an existing system in terms of erosion across the board. That is, incomplete acquisition is the cause of a system that is different from the non-heritage variety (and typically reduced), while the result of attrition is an unsystematic breakdown of the system, eventually leading to total loss of grammatical gender. Some support for our speculation may be found in Schmid's (2002) important work on German Jews in the United States, who had generally also experienced a severe reduction in the use of their L1 over an extended period of time: The occasional mistakes found in gender assignment in the data did not constitute any rule-based reduction in the gender system of their German^6^.

We then turn to the second question, why declensional suffixes are stable in heritage language. The early acquisition of declensional suffixes is generally accounted for by their high frequency and the fact that they are prosodically favored by young children (Anderssen, 2006)^7^. They may also be initially learned as a unit together with the noun, even though they are not considered to be fully acquired until the relevant nouns also appear in appropriate contexts without the suffix. While prosody is unlikely to be a factor in heritage languages, the other two, frequency and chunking, may be responsible for the robustness of the definite forms. That is, highly frequent nouns (such as the ones typically used by our heritage speakers in the corpus) may be stored in memory as units together with the suffix, e.g., *hest****en*** “the horse,” *seng****a*** “the bed,” *hus****et*** “the house.” For this reason, they are easily retrieved, while the indefinite forms must be computed as part of a productive process, e.g., *en hest* “a horse,” *ei seng* “a bed,” *et hus* “a house.” In any case, our heritage data provide further evidence that the definite suffix does not have a gender feature. If this were the case, we would expect these speakers to make a direct link between this form and (other) gender forms: That is, knowing the definite form of a feminine or neuter noun (e.g., *boka* “the book” or *huset* “the house” should make it easy to produce the target-consistent indefinite forms *ei bok* “a book” and *et hus* “a house.” But the data from these heritage speakers show that this is not the case. We therefore conclude that the evidence that we had from acquisition and change from previous studies is now supported by data from a new population.

Finally, we address the third question, why there is no systematic reduction from a three- to a two-gender system in the data of the heritage speakers. In several varieties of Norwegian that have undergone (or are undergoing) a change, the result has been the same: disappearance of the feminine and a development of a two-gender system with common and neuter gender. This has been argued to be partly due to sociolinguistic factors such as language contact or the prestige of the written form *Bokmål* and partly due to the syncretism between masculine and feminine, making it more difficult to distinguish the two in acquisition (e.g., Lødrup, 2011; Trudgill, 2013; Rodina and Westergaard, 2015a). Following up on our speculation above, we would like to suggest that all of these historical developments are due to incomplete acquisition. What we see in our data from the Norwegian heritage speakers, on the other hand, is the result of attrition. If this idea is on the right track, we might have a way to distinguish between the two processes: While incomplete acquisition typically results in a systematic difference between the heritage language and the non-heritage variety, attrition will result in general erosion and considerable variability^8^.

## Conclusion

In this paper, we have presented an investigation of grammatical gender in a corpus of heritage Norwegian spoken in America, the Corpus of American Norwegian Speech (CANS). The corpus consists of data from 50 speakers, whose linguistic profile is as follows: Monolingual Norwegian until age 5–6, English dominant throughout life, and virtually no use of Norwegian in old age. Due to the non-transparency of gender assignment, we expected gender to be vulnerable in this situation of reduced input and use. Based on previous research from acquisition and change, we also expected declensional suffixes to be robust and feminine forms to be more vulnerable than neuter. That is, we expected to find evidence of a reduction in the system, from three genders (masculine, feminine, neuter) to two (common and neuter). Focusing on indefinite articles and possessives, we demonstrated that all three gender forms, masculine, feminine and neuter, are represented in the data. Nevertheless, there is considerable overgeneralization of masculine forms (the most frequent gender forms) in the production of the heritage speakers to both feminine and neuter nouns (as compared with gender in the relevant present-day Norwegian dialects). We also found a substantial difference between the indefinite article (an exponent of gender) and the definite suffixal article (which we consider a declension class marker): While the former is to a large extent affected by overgeneralization, the latter form is virtually always target-consistent. This confirms similar findings from previous research on both acquisition and change. However, we did not find any evidence of a two-gender system in the production of any of the speakers; instead there seems to be overgeneralization of masculine forms across the board. Assuming that the Norwegian of our participants is somewhat attrited, we speculate that this finding is due to a distinction between (incomplete) acquisition and attrition: While the former process typically results in a systematic difference between the heritage language and the non-heritage variety, attrition will lead to general erosion of the system and eventually complete loss of gender.

## Author contributions

All authors listed, have made substantial, direct and intellectual contribution to the work, and approved it for publication.

### Conflict of interest statement

The authors declare that the research was conducted in the absence of any commercial or financial relationships that could be construed as a potential conflict of interest.

## Footnotes

^1^We indicate gender on the noun itself in parenthesis and gender agreement on other targets after a period.

^2^There is only one exception to this, the adjective *liten*∕*lita*∕*lite* “small/little,” which distinguishes between all three genders. This is illustrated in (i).

- (i)
  - en liten gutt
    a.m small.m boy
    “a small boy”
  - ei lita jente
    a.f small.f girl
    “a small girl”
  - et lite hus
    a.n small.n house
    “a small house”

^3^NoTa (Norsk talespråkskorpus—Oslodelen [Norwegian spoken corpus, the Oslo part]), *The Text Lab, Department of Linguistics and Scandinavian Studies*. University of Oslo. Available online at: http://www.tekstlab.uio.no/nota/oslo/index.html

^4^It is not always easy to distinguish loan words from English words that have become an integrated part of American Norwegian speech, e.g., *farmer* or *field*. We have used the following criterion in our selection: All words that currently exist in English and which are pronounced with a clear American pronunciation have been discarded in this paper.

^5^We are grateful to Jan Terje Faarlund for valuable help and discussions concerning this issue.

^6^An important difference between Schmid's (2002) study and ours (pointed out by a reviewer) is that she finds very few non-target-like examples in her data, while there is evidence for considerable erosion in the data of the Norwegian heritage speakers. We would like to suggest that a possible reason for this could be that Schmid's (2002) subjects are first generation immigrants and thus had more robust input in their L1, while the attrition we see in our speakers could have accumulated over 3–4 generations. Furthermore, the German gender system could be said to be somewhat more transparent than the Norwegian one.

^7^Adding a definite suffix to monosyllabic nouns in Norwegian results in a trochaic structure (strong-weak), which is known to be favored by young children (e.g., Gerken, 1994).

^8^A reviewer suggests that our findings could be the result of problems with lexical access in very old speakers rather than attrition. We agree that this could very well be the case—or at least an additional factor. This would predict that also Norwegians living in Norway would experience problems with gender assignment in their old age. Unfortunately, we know of no studies that have investigated this issue, and we therefore have to leave this suggestion to further research.

## Appendix

**Table A1:** **Production of the indefinite article for each of the three genders by all speakers in CANS (*N* = 50)**.

	**M**			**F**			**N**		
**Informant**	**M**	**F**	**N**	**M**	**F**	**N**	**M**	**F**	**N**
**GROUP 1**									
harmony_MN_03gm	5								
harmony_MN_05gm	1								
north_battleford_SK_02gk									
spring_grove_MN_09gm	3								
**GROUP 2**									
billings_MT_01gm	8				2				1
blair_WI_01gm	9				4				
blair_WI_07gm	7				2				
spring_grove_MN_05gm	4				2				
zumbrota_MN_01gk	4				1				1
**GROUP 3**									
blair_WI_02gm	9			2			2		
blair_WI_04gk	16			4					
coon_valley_WI_01gk	5			2			2		
coon_valley_WI_12gm	3						2		
decorah_IA_02gm	5			2			1		
gary_MN_01gm	14						2		
sunburg_MN_04gk	5			4			1		
vancouver_WA_03uk	4			1			1		
westby_WI_02gm	7						2		
**GROUP 4**									
albert_lea_MN_01gk	8			4	8		1		1
chicago_IL_01gk	17			9			6		5
coon_valley_WI_02gm	14			1	3		3	1	4
coon_valley_WI_03gm	29			1	8				1
coon_valley_WI_04gm	6				2		2		2
coon_valley_WI_06gm	36	1			5		1	3	7
coon_valley_WI_07gk	5			1	3		1		
decorah_IA_01gm	15			4			1		2
fargo_ND_01gm	15			5	4		3		
flom_MN_01gm	14			1	12		3		4
flom_MN_02gm	19				10			2	3
gary_MN_02gk	16	1		3	8		1	1	10
glasgow_MT_01gm	4			3			2		3
harmony_MN_01gk	16			2	2		1		1
harmony_MN_02gk	12				4			4	1
harmony_MN_04gm	5				1		1	1	1
north_battleford_SK_01gm	1						4		2
portland_ND_01gm	8			4	1		3		1
portland_ND_02gk	6			8			13		1
rushford_MN_01gm	1	1		1	1				
stillwater_MN_01gm	75			13	25		9		20
sunburg_MN_03gm	13			3	3		4		2
sunburg_MN_12gk	1				1		1		
vancouver_WA_01gm	14			8			2		2
wanamingo_MN_04gk	2			1	1		1		1
webster_SD_01gm	24			3			1		1
webster_SD_02gm	6			4	4				2
westby_WI_01gm	41	1			30		2	1	15
westby_WI_03gk	13				4			3	6
westby_WI_05gm	3						3	3	
westby_WI_06gm	9	1		3	1		2		
zumbrota_MN_02gm	4			1	4				

The baseline is the Nynorsk dictionary adjusted for some typical patterns in Eastern Norwegian dialects. Group 1, Gender system unclear; Group 2, Possibly a three-gender system; Group 3, Masculine forms only; Group 4, Mixture of gender forms.
